# The Secretome and *N*-Glycosylation Profiles of the Charophycean Green Alga, *Penium margaritaceum*, Resemble Those of Embryophytes

**DOI:** 10.3390/proteomes6020014

**Published:** 2018-03-21

**Authors:** Eliel Ruiz-May, Iben Sørensen, Zhangjun Fei, Sheng Zhang, David S. Domozych, Jocelyn K. C. Rose

**Affiliations:** 1 Plant Biology Section, School of Integrative Plant Science, Cornell University, Ithaca, NY 14853, USA; eliel.ruiz@inecol.mx (E.R.-M.); is265@cornell.edu (I.S.); 2 Red de Estudios Moleculares Avanzados, Instituto de Ecología A. C., Cluster BioMimic, Carretera Antigua a Coatepec 351, Congregación el Haya, CP 91070 Xalapa, Veracruz, Mexico; 3 Boyce Thompson Institute, Ithaca, NY 14853, USA; zf25@cornell.edu; 4 U.S. Department of Agriculture-Agricultural Research Service, Robert W. Holley Center for Agriculture and Health, Ithaca, NY 14853, USA; 5 Institute of Biotechnology, Cornell University, Ithaca, NY 14853, USA; sz14@cornell.edu; 6 Department of Biology and Skidmore Microscopy Imaging Center, Skidmore College, Saratoga Springs, NY 12866, USA; ddomoz@skidmore.edu

**Keywords:** secretome, cell wall proteins, embryophytes, charophycean green algae, *N*-glycosylation

## Abstract

The secretome can be defined as the population of proteins that are secreted into the extracellular environment. Many proteins that are secreted by eukaryotes are *N*-glycosylated. However, there are striking differences in the diversity and conservation of *N*-glycosylation patterns between taxa. For example, the secretome and *N*-glycosylation structures differ between land plants and chlorophyte green algae, but it is not clear when this divergence took place during plant evolution. A potentially valuable system to study this issue is provided by the charophycean green algae (CGA), which is the immediate ancestors of land plants. In this study, we used lectin affinity chromatography (LAC) coupled with mass spectrometry to characterize the secretome including secreted *N*-glycoproteins of *Penium margaritaceum*, which is a member of the CGA. The identified secreted proteins and *N*-glycans were compared to those known from the chlorophyte green alga *Chlamydomonas reinhardtii* and the model land plant, *Arabidopsis thaliana*, to establish their evolutionary context. Our approach allowed the identification of cell wall proteins and proteins modified with *N*-glycans that are identical to those of embryophytes, which suggests that the *P. margaritaceum* secretome is more closely related to those of land plants than to those of chlorophytes. The results of this study support the hypothesis that many of the proteins associated with plant cell wall modification as well as other extracellular processes evolved prior to the colonization of terrestrial habitats.

## 1. Introduction

Cells secrete populations of proteins collectively referred to as the ‘secretome’ for purposes such as maintaining and modulating cell and tissue integrity, regulating the external environment, and defense [1,2,3,4,5]. The composition of the secretome varies among the kingdoms of life and consists of a few hundred up to more than a thousand proteins [3,6,7,8], with the specific populations being highly dynamic and adaptive to different environments and stress conditions [3,4,7]. In this regard, the secretome of an organism can reflect a particular habitat including the complexity of the organism’s life cycle and its lifestyle [3]. For instance, a significant proportion of the secretome of land plants includes proteins associated with the assembly and remodeling of complex polysaccharide cell walls [9,10,11,12], which reflects the importance of cell wall modification during cell growth, differentiation, and responses to different stress conditions. Other sets of proteins contribute to wall reinforcement and the deposition of structural protein frameworks, phenylpropanoid polymers such a lignin or lipid barriers in the cuticle of epidermal cells. Moreover, during pathogen infection, land plants produce large numbers of hydrolytic and other degradative enzymes as well as other defense-related proteins to reinforce the host cell wall and suppress microbial infection [13,14]. Notably, much of the land plant secretome is devoted to surviving in a terrestrial environment with its attending challenges of biotic and abiotic stresses and establishing a supportive and structurally plastic, cell wall infrastructure. 

In contrast, the secretome of the chlorophyte green alga *Chlamydomonas reinhardtii*, which lives in an aquatic habitat, is mainly composed of a variety of hydroxyproline-rich glycoproteins (HRGPs), flagellar proteins, and peptidases [15,16,17,18]. The HRGPs form a relatively simple cell wall matrix and are also associated with promoting the adhesion of the gametes during mating [19,20]. The absence of complex polysaccharides in the cell wall of *C. reinhardtii* mirrors a relative paucity of cell wall associated proteins in its secretome [17,18,21].

**Figure 1:**
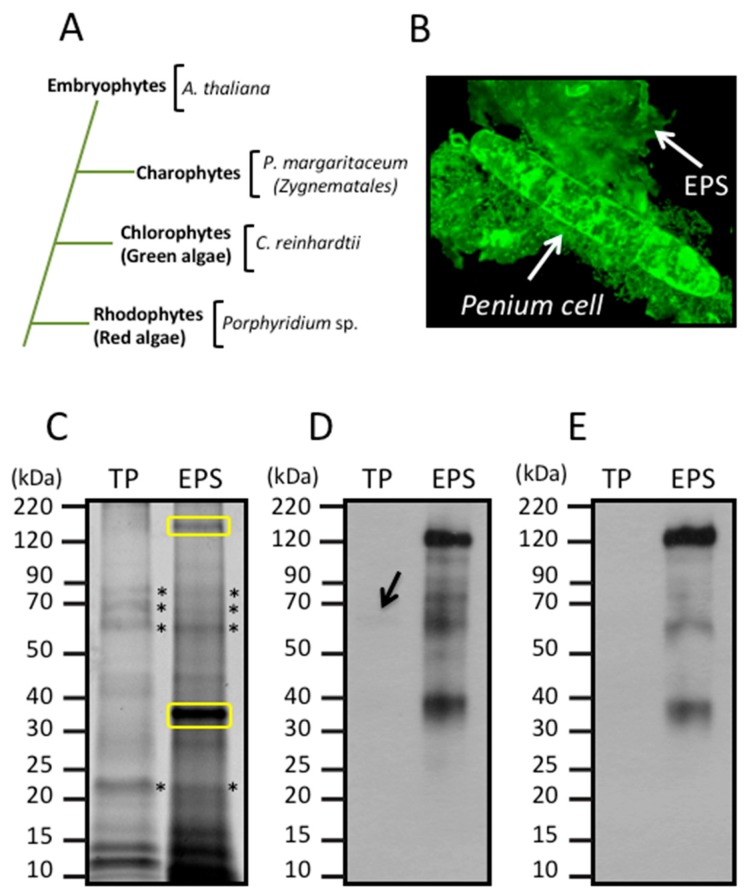
(**A**) Phylogenetic relationship between Embryophytes, Charophytes, Chlorophytes, and Rhodophytes based on [54] and (**B**–**D**) immunological detection of extracellular polymeric substance (EPS), β-1,2-xylose, and α-1,3-fucose in *Penium margaritaceum* cultures. (**B**) Live cell immunodetection of EPS using an anti-EPS antibody. The EPS accumulates on the surface of the cell-wall and surrounding milieu, scale bar = 8 μm. (**C**) Proteins extracted from *P. margaritaceum* cells (total protein, TP) or EPS were fractionated by SDS-PAGE and stained with SYPRO Ruby. Asterisks indicate protein bands observed in both TP and EPS. Yellow boxes indicate the densest protein bands in the EPS protein extract. Numbers to the left of each gel show the molecular weight in kDa of the protein markers in the 1D SDS-PAGE. Immunological analysis of TP and EPS extracts using antibodies raised against epitopes of β-1,2-xylose (**D**) and α-1,3-fucose (**E**). In (**D**), the arrow indicates the weak immunodetection of β-1,2-xylose epitopes.

A large proportion of the proteins in eukaryotic secretomes are *N*-glycosylated. A post-translational modification (PTM) that exhibits a substantial degree of structural diversity between different kingdoms [22,23,24]. Much attention has been paid to the *N*-glycosylation of proteins that are secreted by several microalgae [17,25] in part due to an interest in the potential use of these proteins by the pharmaceutical industry [25]. In this regard, and in contrast to the *N*-glycan structures of land plants [26], structural studies of cell wall glycoproteins from the red microalgae, *Porphyridium* sp., revealed the presence of a specific *N*-glycan structure with internal 6-*O*-methylated mannose residues and a xylose residue attached to an external mannose residue of the 6-antenna [27]. In addition, structural studies showed that the diatom, *Phaeodactylum tricornutum*, produces predominantly high mannose type *N*-glycans and less frequently *N*-glycans with α-1,3-fucose (Fuc) residues [25]. It has also been reported that sialylated *N*-glycans similar to those commonly found in mammalian glycoproteins are present in *C. reinhardtii* [28,29]. *N*-glycans from *C. reinhardtii* are partially methylated on the outer mannose residues and contain the β-1,2-xylose residues typically found in the *N*-glycans of land plants [17]. Furthermore, unlike red microalgae, land plants commonly produce extracellular glycoproteins containing complex type *N*-glycans decorated with β-1,2 xylose and α-1,3-fucose [30]. *C. reinhardtii* diverged from land plants over one billion years ago but has retained many genes of the last common plant-animal ancestor [21] and, accordingly, its *N*-glycosylation machinery shows both differences and similarities to that of land plants. However, little is known about the evolutionary transition where the *N*-glycan structures commonly found in land plants diverged from typical algal *N*-glycans (see Figure 1A). To this end, the charophycean green algae (CGA) represent an interesting model since they are considered to be the closest living ancestors of land plants (or embryophytes) and extant examples are likely descendants of the green plants that first colonized terrestrial habitats about 450 million years ago [31,32,33].

The CGA consists of six main groups including the Charophytales, the Coleochaetales, the Zygnematales, the Klebsormidiales, the Chlorokybales, and the Mesostigmatales of which the three latter are considered to be early diverging and the three former to be later diverging [33]. There is no clear consensus as to which of the later diverging groups was sister to land plants, but the Zygnematales [34,35] of which *Penium margaritaceum* is a member, has recently been proposed as a strong candidate.

The application of high throughput technologies to study species within the CGA either to identify cell wall genes or to characterize cell wall components has shed light on the evolutionary history of the plant cell wall. For example, analysis of expressed sequence tags and RNA-Seq datasets from CGA species has revealed genes associated with cell wall biogenesis with considerable sequence similar to those of land plants [36,37]. Furthermore, the recently published genome sequence of *Klebsormidium flaccidum*, a member of the CGA, has suggested the presence of genes associated with cell wall formation and modification including those encoding polygalacturonase, cellulose synthase, 3-beta-d-glucan synthase, and pectate lyase [38]. These observations are supported by glycan microarray analysis of extracted cell wall polysaccharides and sugar linkage analysis, which showed that later diverging CGA contain polymers commonly found in embryophytes [37] as well as numerous immunocytochemical studies of diverse CGA species [39,40,41,42,43]. Among the CGA, *P. margaritaceum*, has shown great potential for fundamental studies associated with cell wall biogenesis [41,42] including the secretion of cell wall proteins. Live cell labeling of *P. margaritaceum* cells using monoclonal antibodies that recognize polysaccharide epitopes of land plant cell walls also allows the study of cell wall dynamics [41]. An additional feature of *P. margaritaceum* is that it produces and secretes an extracellular polymeric substance (EPS) that covers the outer surface of the cell wall. This EPS has been associated with establishing biofilm communities and for providing a barrier against biotic or abiotic stress [44,45,46,47,48,49]. One of the most notable functions of the EPS is protection against desiccation [47], which was likely a key factor for the transition from fresh water to terrestrial environments [39,44,45,46,49,50,51,52]. It is also of note that *P. margaritaceum* is the only CGA species to date that has been stably transformed and, therefore, could lend itself to functional characterization of genes encoding secreted proteins and those involved in *N*-glycosylation [53]. 

Recently, multi-lectin affinity chromatography (MLAC) has been used for the systematic characterization of the cell wall proteome/secretome of several eukaryotes including the model plants *Arabidopsis thaliana* and tomato (*Solanum lycopersicum*) [6,30,55]. However, in contrast to land plants, little is known regarding the secretory pathway of the CGA or whether the pattern of *N*-glycosylation is similar to that of land plants. However, a comparative bioinformatics study of *K. flaccidum* has suggested the presence of homologs of many of the genes involved in *N*-glycosylation in land plants [56]. We hypothesized that characterization of the CGA secretome and structural analysis of the constituent *N*-glycans might provide insights into the evolution of *N*-glycosylation and the plant secretome and reveal whether it is more similar to that of the chlorophytes or embryophytes and whether any unique patterns are evident in the CGA. In this study, we used Concanavalin A-based lectin affinity chromatography (LAC) coupled with mass spectrometry to characterize the secretome of *P. margaritaceum*. We also identified proteins associated with the EPS and determined the structural features of the *N*-glycans of secreted *N*-glycoproteins. 

## 2. Material and Methods 

### 2.1. P. margaritaceum Growth Conditions

*Penium margaritaceum* Brébisson (Skidmore College Algal Culture Collection) was maintained in sterile liquid cultures of Woods Hole (WH) medium (pH 7.2) [57] at 18 ± 2 °C in a photoperiod of 16 h light/8 h dark with 74 μM photons m^−2^ s^−1^ Photosynthetic Photon Flux of cool white fluorescent light. The cells were sub-cultured every week and cells/culture medium from log-phase cultures (14–21 d old cultures) were harvested for subsequent experiments.

### 2.2. Collection of P. margaritaceum Cells and EPS

Cells were collected in sterile 50 mL centrifuge tubes and centrifuged for 1 min at 1000× *g*. The supernatants consisting of culture medium containing secreted EPS were collected and stored on ice. The cell pellets were re-suspended in fresh WH, vortexed vigorously for 30 s, and re-centrifuged. This washing step was repeated three times. The supernatants were combined and stored on ice. The cell pellets were combined and used for subsequent protein extractions.

For EPS collection, cell supernatants were placed in sterile 1 L, non-tissue culture treated plastic flasks, and vigorously shaken for 2 min. The supernatants were then centrifuged for 10 min at 12,000× *g*. The resultant supernatant contained large floating masses of EPS gel. These masses were collected using a sterile pipette and then combined and used for protein extraction.

### 2.3. Algal Protein Extraction and LAC

Five mL of concentrated *P. margaritaceum* culture was mixed with 10 mL of extraction buffer (25 mM Tris, pH 7, 0.2 M CaCl_2_, 0.5 M NaCl, and 20 μL/g fresh weight protease inhibitor cocktail (Sigma-Aldrich, St. Louis, MO, USA)). The cells were then disrupted using a Bead-Beater homogenizer and 0.5 mm zirconia/silica beads for 5 min as previously described [58]. The resulting suspension was sonicated 10 times for 10 s with a sonic dismembrator model 100 (Fisher Scientific, Inc., Pittsburgh, PA, USA) on full power. The suspension was centrifuged at 25,000× *g*, at 4 °C for 30 min and the supernatant was used for enrichment of *N*-glycoproteins by LAC as previously reported [59]. Briefly, 5 mL ConA cartridges (Qiagen, Hilden, Germany) were pre-equilibrated with binding buffer (20 mM Tris-HCl pH 7.0, 0.5 M NaCl, 1 mM CaCl_2_, 1 mM MnCl_2_, and 1 mM MgCl_2_), at a flow rate of 0.08 mL/min. The supernatants from the crude protein extracts described above were loaded onto a ConA column using a fast protein liquid chromatography (FPLC) system (Pharmacia Fine Chemicals AB, Uppsala, Sweden) and, after washing the column with three column volumes of binding buffer, the bound proteins were eluted with five column volumes of elution buffer (binding buffer containing 0.5 M α-methyl-d-mannopyranoside (Calbiochem, La Jolla, CA, USA)) at a flow rate of 0.75 mL/min. The eluted glycoproteins were concentrated and the elution buffer was exchanged for 100 mM ammonium bicarbonate by using a 5 kDa cutoff centrifugal concentrator (Amicon Ultra-15, Millipore, Billerica, MA, USA) before being lyophilized in a Freezone 6 freeze dry system (Labconco, Kansas City, MO, USA).

### 2.4. EPS Protein Extraction 

A total of 50 mL of EPS was mixed with 100 mL of extraction buffer and placed on a rocking table for 2 h at 4 °C before filtering through a 0.45 μm Acrodisc syringe filter (Pall, Newquay Cornwall, UK). The volume of the solution was then reduced to 5 mL using a 5 kDa cutoff centrifugal concentrator (Amicon Ultra-15, Millipore, Billerica, MA, USA), and the solvent was exchanged three times by adding equal volumes of 100 mM ammonium bicarbonate. The volume was reduced by 50% using centrifugation according to the manufacturer’s instructions. The final sample was frozen in liquid nitrogen, stored at −80 °C, and lyophilized as above.

The protein concentration was determined using a BCA™ Assay Kit (Pierce; Rockford, IL, USA) with bovine serum albumin (BSA) as a standard. To visualize the protein composition of the crude extracts, aliquots (10 μg) were mixed with 5X Laemmli buffer [60], fractionated on 12% Tris-glycine SDS-PAGE gels (TGX™ gels, BioRad; Hercules, CA, USA) and stained with SYPRO ruby (Life Technologies, Grand Island, NY, USA).

### 2.5. Trypsin Digestion and N-glycopeptide Enrichment

Trypsin digestion and *N*-glycopeptide enrichment were carried out as previously described with slight modifications (see Figure S1) [30]. Briefly, *N*-glycoproteins enriched by LAC and EPS proteins lyophilized samples were dissolved in 8 M urea in 100 mM ammonium bicarbonate, reduced with 10 mM DTT for 1 h, and alkylated with 25 mM iodoacetamide in the dark for 30 min at room temperature before dilution to a final concentration of 1 M urea with 100 mM ammonium bicarbonate. Proteins were digested with trypsin (Trypsin Gold, Mass Spectrometry Grade, Promega, Madison, WI, USA) at a 1:20 *w*/*w* trypsin concentration with a protein ratio for 16 h at 37 °C. The pH of the trypsinized samples was then adjusted to 5.0 with 0.1% trifluoroacetic acid (TFA). EPS tryptic peptides were desalted with C18 solid phase extraction cartridge (Waters, Milford, MA, USA) and dried with a SpeedVac (Thermo Savant, Holbrook, NY, USA) and analyzed by nanoLC-MS/MS. Tryptic peptides from *N*-glycoproteins enriched by LAC were loaded onto a porous graphite carbon (PGC) cartridge (Thermo Scientific, Bellefonte, PA, USA). Subsequently, 1 mL wash solvent (5% acetonitrile (ACN) in MilliQ water with 0.1% TFA (*v*/*v*)) was passed through the column. The flow through was recovered and desalted with a C18 solid phase extraction cartridge (Waters, Milford, MA, USA). The bound *N*-glycopeptides were eluted with 1 mL elution solvent (50% ACN in MilliQ water with 0.1% TFA (*v*/*v*)). Both flow through and bound *N*-glycopeptides were dried using a SpeedVac (Thermo Savant, Holbrook, NY, USA) prior to nanoLC-MS/MS analysis.

### 2.6. NanoLC-MS/MS (Liquid Chromatography-Tandem Mass Spectrometry) Analysis

The EPS, PGC bound and flow through samples were analyzed by nanoLC-MS/MS using an LTQ-Orbitrap Velos (Thermo-Fisher Scientific, San Jose, CA, USA) mass spectrometer equipped with a “CorConneX” nano ion source (CorSolutions LLC, Ithaca, NY, USA) using a 10-μm analyte emitter (NewObjective, Woburn, MA, USA). The Orbitrap was interfaced with an UltiMate 3000 RSLC system (Dionex, Sunnyvale, CA, USA). Each reconstituted fraction (5 μL) was injected onto a PepMap C18 trap column (5 μm, 300 μm × 5 mm, Dionex) at 20 μL/min flow rate and then separated on a PepMap C-18 RP nano column (3 μm, 75 μm × 15cm) using a 60-min gradient from 5% to 40% ACN in 0.1% formic acid (FA) at 300 nL/min. The Orbitrap was operated in positive ion mode with nanospray voltage set at 1.5 kV and source temperature at 275 °C. The Fourier Transform mass analyzer (FTMA) was calibrated using either the background polysiloxane ion signal (*m*/*z* 445.120025) as an internal lock mass or the Ultramark 1621 external calibrant (catalog number: 88323, Thermo Fisher Scientific™ Pierce™). The instrument was operated in parallel data-dependent acquisition (DDA) mode using the FTMA for one survey MS scan at a resolution of 60,000 (FWHM at *m*/*z* 400) for the mass range of *m*/*z* 375–1800 for precursor ions, which was followed by MS/MS scans of the seven most intense peaks with multiple charged ions above a threshold ion count of 7500 in both LTQ mass analyzer and higher-energy collisional dissociation (HCD)-based FTMA at 7500 resolution. Dynamic exclusion parameters were set at a repeat count of 1 with a 25 s repeat duration, exclusion list size of 500, 45 s exclusion duration, and ±10 ppm exclusion mass width. Collision induced dissociation (CID) parameters were set at an isolation width 2.0 *m*/*z*, normalized collision energy 35%, activation Q at 0.25, and activation time 10 ms. The activation time was 0.1 ms for HCD analysis. All data were acquired with the Xcalibur 2.1 software (Thermo-Fisher Scientific, Waltham, MA, USA). 

The nanoLC-MS/MS analysis for characterization of glycosylation sites was performed on an UltiMate3000 nanoLC system (Dionex, Sunnyvale, CA, USA) coupled with a hybrid triple quadrupole linear ion trap mass spectrometer, the 4000 Q Trap (AB SCIEX, Framingham, MA, USA). The tryptic peptides bound to PGC (5 μL) were injected. MS data acquisition in a 4000 Q trap was performed using Analyst 1.4.2 software (Applied Biosystems, Foster City, CA, USA) and operated using precursor ion (PI) scan-triggered information-dependent acquisition (IDA) analysis. The precursor ion scan of the oxonium ion (HexNAc^+^ at *m*/*z* 204.08) was monitored using a step size of 0.2 Da across a mass range of *m*/*z* 500–1600 and the parameters were set as previously reported [61]. For IDA analysis, each precursor ion scan was followed by one enhanced resolution scan and the two highest intensity ions with multiple charge states were selected for MS/MS using rolling collision energy (CE) that was set based on the charge state and *m*/*z* value of each ion. In the rolling CE setting, high CE and low CE were applied in two separate acquisition methods to gain sequence information regarding both peptides and glycans. We obtained MS/MS spectra with cross-ring fragmentation using high energy. However, the glycan structures reported in this study are based on empirical glycan composition and structure of *N*-glycans reported previously in the literature [61].

### 2.7. Data Analysis and Interpretation 

DDA raw data from the Orbitrap was converted into MASCOT generic format (MGF) files using Proteome Discoverer 1.3 (PD1.3, Thermo, Waltham, MA, USA). The subsequent searches were carried out using Mascot Daemon (version 2.3, Matrix Science, Boston, MA, USA) with the following parameters including semi-tryptic protease specificity with one missed cleavage allowed, 20 ppm precursor mass tolerance, 0.8 Da for CID and 0.05 Da for HCD fragment ion mass tolerance with a fixed modification of cysteine carbamidomethylation, and variable modifications of methionine oxidation and asparagine/glutamine deamidation. Mass spectra were used to search a translated in-house unigene databases generated from RNA-Seq data derived from *P. margaritaceum* cell cultures or PlantGDB (http://www.plantgdb.org/). In addition, we used a unigene database generated with sequence reads from *Coleochaete orbicularis* and *Spirogyra pratensis* [36]. Only peptides that matched with a MASCOT score above the 95% confidence interval threshold (*p* < 0.05, MASCOT score ≥44) were considered for protein identification. In cases where the protein was identified by a single peptide match, the threshold was set at a 99% confidence interval (MASCOT score ≥51). These MASCOT scores resulted in a false-positive identification rate of 4.56%. Only proteins containing at least one unique peptide, which is a sequence that had not been previously assigned to a different protein, were considered. 

### 2.8. Live Cell and EPS Labeling of P. margaritaceum 

Cells from 14 day old cultures were collected and washed as described above. The pellet was re-suspended in fresh WH, lightly shaken, and then 25 μL aliquots of cell suspension were placed in the wells of 10-welled poly-l-lysine coated immuno slides (EMS, Ft. Washington, PA, USA). The cells were allowed to settle for 5 min and extra growth medium was suctioned off. A 20 μL drop of fresh WH was placed on the cells and the slides were allowed to sit under the aforementioned growth conditions (see Section 2.1) for 10 min. The cells were then labeled for immunofluorescence imaging with an anti-EPS antibody (anti-Skid8) using a previously described protocol [39,40]. The secondary antibody was TRITC-conjugated goat anti-rat antibody (Sigma). After the final wash, a cover slip was mounted onto the slide and cells were observed using an Olympus Fluoview 300 confocal laser scanning microscope.

### 2.9. Bioinformatic Analysis

RNA-Seq raw data is available with the accession number SRP133135 in GenBank (https://www.ncbi.nlm.nih.gov/sra/SRP133135), and unigenes associated with identified proteins are provided with the accession number GGHW00000000.1 (https://www.ncbi.nlm.nih.gov/Traces/wgs/?val=GGHW01). Functional annotation of the proteins was carried out using Blast2GO software version 5.0.8 (www.blast2go.com), UniProt functional annotation (http://www.uniprot.org/), and following the procedures outlined in Jamet et al. [62]. Proteins were screened for the predicted presence of an *N*-terminal endoplasmic reticulum (ER) targeting signal peptide (SP) using the Signal P 4.1 program [63]. Those with a predicted SP were analyzed for the presence of predicted *N*-glycosylation sites using the NetGlyc 1.0 server (http://www.cbs.dtu.dk/services/NetNGlyc/). PSORT [64], Target P [65], and PredAlgo [66] were used for determining the predicted subcellular locations of the identified proteins. The TMHMM Server v. 2.0 [67] and SOSUI [68] were used to predict transmembrane domains. For the secretome comparison, we used the complete proteome sequences of the *A. thaliana* (TAIR10, http://www.arabidopsis.org/wublast/index2.jsp), *Volvox carteri* (a chlorophyte, http://genome.gi.doe.gov/pages/blast.jsf?db=Volca) and *C. reinhardtii* (v4.0, http://genome.jgi-psf.org/pages/blast.jsf? db=Chlre4) proteomes. The mass spectrometry proteomics data have been deposited to the ProteomeXchange Consortium via the PRIDE partner repository with the dataset identifier PXD009123 (https://www.ebi.ac.uk/pride/archive/).

## 3. Results

### 3.1. Glycan Structures of P. margaritaceum Extracellular N-glycoproteins 

*P. margaritaceum* cells actively produce and secrete large amounts of EPS, which can be visualized using polyclonal antibodies specifically raised against this adhesive mucilage. It accumulates on the surface of the cell as well as in the surrounding media/substrate (see Figure 1B). This can be separated from the cells by manually shaking the cultures and subsequent centrifugation. Total protein (TP) from the cells as well as proteins extracted from the EPS were visualized on an SDS-PAGE gel stained with SYPRO Ruby (see Figure 1C). Specific protein patterns were visualized in each sample with two major bands of around 38 kDa and about 180 kDa exclusively observed in the EPS fraction. Other bands of similar molecular weight to each other were observed in both samples (see Figure 1C). 

In green plants, β-1,2-xylose (Xyl) and α-1,3-fucose (Fuc) residues, are commonly found in *N*-glycoproteins [26] (see Figure 1A). In order to determine the presence of these sugar residues in the *P. margaritaceum* glycoproteins, we carried out an immune detection assay with antisera that recognize Xyl (see Figure 1D) and Fuc residues (see Figure 1E). This resulted in a barely detectable signal in the TP sample (Figure 1D) while a strong signal was seen in proteins extracted from the EPS (Figure 1D,E). This suggests the presence of highly glycosylated *N*-glycoproteins with Xyl and Fuc residues in the *P. margaritaceum* EPS sample.

### 3.2. Secretome Profiling by LAC and nanoLC-MS/MS 

**Figure 2:**
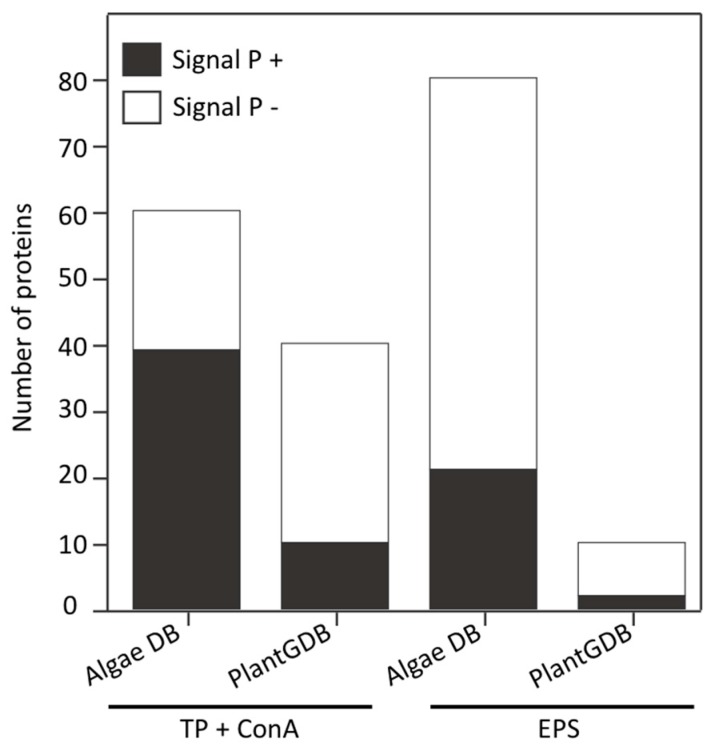
Proteins identified by nanoLC-MS/MS using a *Penium margaritaceum*, *Coleochaete orbicularis*, and *Spirogyra pratensis* database derived from RNA-Seq data or PlantGDB (http://www.plantgdb.org/). The presence of signal peptides was predicted using the Signal P 4.1 program [63]. TP: total proteins extracted from *P. margaritaceum* cells were used for the enrichment of glycoproteins with Concanavalin A (ConA). The extracellular polymeric substance (EPS) was analyzed without lectin enrichment.

ConA lectin, which we used as an affinity matrix in this study, specifically binds α-mannose/α-glucose on *N*-glycans and it has little cross-reactivity with non-glycosylated proteins. However, it should be noted that since it binds to specific *N*-glycan structures, it does not recognize all glycoproteins [69]. Proteins that bound to the ConA lectin column were digested with trypsin and the resulting glycopeptides enriched with PGC and flow through were analyzed by nanoLC-MS/MS. Peptide sequences derived from the resulting spectra were used to search an in-house *P. margaritaceum* unigene database derived from RNA-Seq data or the green plant database (PlantGDB, http://www.plantgdb.org). We identified 57 proteins using the *P. margaritaceum* RNA-Seq library while a search of a unigene sequence database of *C. orbicularis* and *S. pratensis* [36] allowed the identification of three additional proteins (see Table S1). Computational predictions using Signal P 4.1 indicated that 39 (65%) of the identified proteins contained secretory signal peptides (see Figure 2 and Table S1). In contrast, matching the spectra to the PlantGDB resulted in the identification of 40 proteins of which 10 (25%) were predicted to contain signal peptides (see Figure 2 and Table S2). The *P. margaritaceum* database was found to be more effective for identifying proteins and subsequent analysis was performed exclusively using protein sequences identified using this database. 

Computational analysis also predicted that 30 (50%) of the identified proteins contained the canonical *N*-glycosylation N-X-S/T sequon and that 19 of them had more than two glycosylation sites (see Table S1). We used PSORT and Target P to predict the subcellular location of the identified proteins and 39 were predicted to be localized in the secretory pathway while 21 had other predicted subcellular locations (see Table 1 and Table S1). Further functional classification of the identified proteins based on sequence homology using the Blast2GO software (www.blast2go.com), the UniProt functional annotation database (http://www.uniprot.org/), and procedures outlined in Jamet et al. [62] revealed cell wall associated proteins, proteases, and oxido-reductases. Interestingly, homologs of some of the cell wall associated proteins identified in *P. margaritaceum* have been found in secretome studies of land plants [6,55,59,70]. For example, we identified a leucine-rich repeat extensin-like protein 1 (LRX1, Q9LJ64), an arabinogalactan protein (AGP, Q9M7I5), and a fasciclin-like arabinogalactan protein (FLA, G7ILU2), which suggests the presence of these types of cell wall associated proteins prior to the emergence of terrestrial plants. We also identified several protein disulfide isomerase proteins (E3W9C1, Q9FEG4 and Q8VX13, Table S1), which are often annotated as residing in the ER or vacuole. 

### 3.3. Identification of Proteins Associated with the EPS 

Proteins extracted from the EPS were digested with trypsin and analyzed by nanoLC-MS/MS using an LTQ-Orbitrap Velos mass spectrometer. The *P. margaritaceum* database also proved to be the best option for protein identification in the EPS sample (see Figure 2, Tables S3 and S4). We identified 75 proteins using the *P. margaritaceum* database and five additional proteins using unigene sequence from *C. orbicularis* and *S. pratensis*. Of these, 21 (26%) were predicted to contain a secretory signal peptide (see Figure 2 and Table S3). Homologs of many of these proteins have been found in the secretome of embryophytes [6,55,59,70] and some of them, such as the structural constituent of cell wall (B9S9J6) and formin-like protein 2 (Q7XUV2), have a direct association with the cell wall (see Table 1 and Table S1) [71]. Several proteins identified in the EPS sample were predicted to contain *N*-glycosylation sites and four *N*-glycoproteins identified in the EPS were also identified in the sample of ConA-enriched glycoproteins (Q9FEG4, Q9M2R9, F4JJL3, and A9RQJ8, Table 1). This is consistent with the strong signal observed in the immunological analysis of the EPS protein extracts with antibodies recognizing the (Xyl) and (Fuc) residues of *N*-glycoproteins (see Figure 1D,E). In addition to the secreted proteins, the EPS extracts were shown to contain a high percentage (74%) of predicted intracellular proteins, which might reflect cell lysis in culture.

### 3.4. Land Plant Cell Wall Protein Homologs in the Cell Wall of P. margaritaceum 

**Figure 3:**
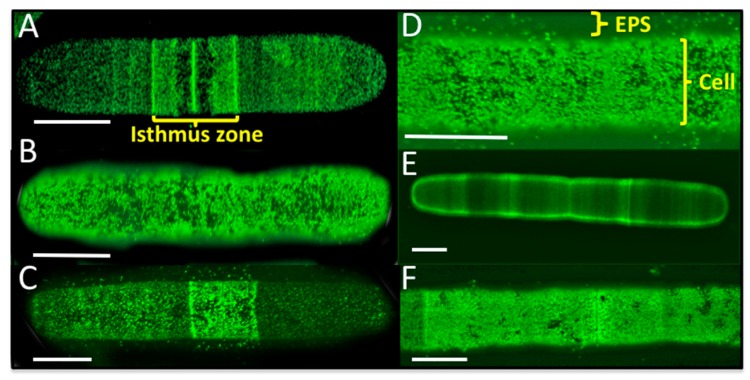
Live cell labeling of *Penium margaritaceum* cultures using monoclonal antibodies raised against arabinogalactan proteins, JIM8 (**A**), JIM13 (**B**), JIM16 (**C**,**D**), and extensin JIM20 (**E**,**F**), scale bars = 15 μm.

The identification of structural cell wall proteins in *P. margaritaceum* that are similar to those found in land plants such as arabinogalactan protein (AGP; Q9M7I5), fasciclin-like arabinogalactan protein (FLA; G7ILU2), and leucine-rich repeat/extensin (LRX1; Q9LJ64) indicates some similarities in the structural composition of the primary cell walls of land plants and CGA. AGPs and extensins are glycoproteins that have been proposed to be covalently cross-linked to the polysaccharide matrix in the walls of land plants [72,73,74]. To confirm the presence and localization of these structural cell wall glycoproteins in *P. margaritaceum*, we carried out live cell labeling using monoclonal antibodies raised against AGP (JIM8, JIM13, and JIM16) and extensin (JIM20) polysaccharide epitopes [75,76,77,78]. Live cell imaging of *P. margaritaceum* labeled with monoclonal antibodies recognizing AGP epitopes, showed an intense punctuated pattern either on the surface of the cell (see Figure 3A–D) or more loosely associated with the cell wall/EPS (see Figure 3C,D) with more intense signals running along the center of the cell (see Figure 3A,C) around the isthmus zone. This suggests that AGPs are secreted and accumulate on the cell surface and possibly in the extracellular medium of *P. margaritaceum*. Immune detection of the extensin epitope recognized by JIM20 showed several areas along the cell with a stronger signal (see Figure 3E) and a dense punctuate pattern covering almost the whole cell surface (see Figure 3F). Taken together, the proteomic identification and immune-labeling assays provide strong evidence that AGPs and extensins are present in the primary cell wall of *P. margaritaceum*, which is consistent with an earlier study based on immunological evidence [37].

### 3.5. Structural Determination of N-glycosylation Patterns in the P. margaritaceum Secretome

**Figure 4:**
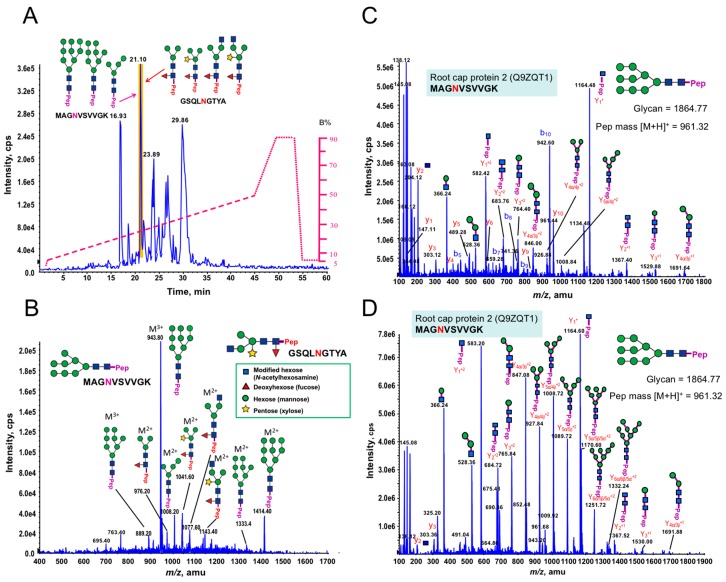
Identification of *N*-linked glycopeptides from *Penium margaritaceum* cultures by precursor ion scan triggered data-dependent (PI-IDA) MS/MS analysis. (**A**) A base peak chromatogram of an LC-MS precursor ion scan (on *m*/*z* +204.08) triggered IDA for enriched tryptic glycopeptides from *P. margaritaceum* proteins. Pep in purple and red indicates glycoforms associated with MAGNVSVVGK and GSQLNGTYA, respectively. (**B**) A representative MS spectrum acquired from a peak eluted at 21.1 min (see Figure 4A). The spectrum shows a typical “high mannose” and “complex type” *N*-glycan including pentose (β-1,2-xylose) and/or deoxyhexose (α-1,3-fucose) linked to the core Man_3_GlcNAc_2_ structure. (**C**) An MS/MS spectrum of an *m*/*z* 942.69^3+^ ion, which reveal a “high mannose” *N*-linked glycopeptide (MAGNVSVVGK) by using high energy fragmentation (at CE 52). The high energy CID fragmentation yielded a complete y-ion/b-ion series along with many y-ion series (up to four mannose residues), which resulted in confident identification of the peptide MAGNVSVVGK from root cap protein 2 (Q9ZQT1) containing nine mannose residues. (**D**) An MS/MS spectrum of same *m*/*z* 942.69^3+^ ion using low energy fragmentation (at CE 34) yielded a complete y-ion series allowing the confident determination of the glycan structure (as a nine-mannose “high mannose” glycan) in the glycopeptide MAGNVSVVGK from the root cap protein 2 (Q9ZQT1).

Most cell wall associated proteins are highly glycosylated. AGPs, FLAs, and LRX1 are commonly *O*-glycosylated on serine (S) or hydroxyproline (Hyp) residues [79,80,81]. In addition LRX1 and FLAs contain the consensus sequence N-X (P)-S/T for *N*-glycosylation [73]. Our data indicates the presence of putative glycoproteins in the *P. margaritaceum* secretome. However, since nothing is known about protein glycosylation in any CGA species, we further analyzed our proteins enriched by ConA to determine the structures of the *N*-glycans. Interestingly, we found structures very similar to those found in embryophytes as the MS/MS spectra of the *N*-linked glycosylation of glycopeptides showed the presence of Xyl and Fuc residues commonly found in complex *N*-glycans in embryophytes (see Figure 4 and Figure S2) [55,70]. We identified three glycoforms of the peptide MAG**N**VSVVGK that were distributed over two different charge states by precursor ion scan triggered data-dependent MS/MS analysis with high and low collision energy, which is shown in Figure 4B,C, respectively. This glycopeptide corresponds to the maize root cap protein 2 (Q9ZQT1). These glycoforms had four, eight, and nine mannose residues and all corresponded to the high mannose type (see Figure 4A and Table 2). In addition, at the same elution time, we found a second *N*-glycopeptide (GSQLNGTYA) with four glycoforms that contained either Xyl or Fuc or both residues (see Figure 4A). These glycoforms corresponded to the receptor of activated protein kinase C1 (Table 2, A8J8Y1). Six additional *N*-glycan structures with Xyl and Fuc residues were identified, but their corresponding peptide sequences could not be resolved (see Table 2 and Figure S2). In addition, the low concentration of *N*-glycoproteins in the EPS did not allow for structural determination of any *N*-glycans. However, the combination of immune-labeling (Figure 1D,E) and MS analysis of the enriched *N*-glycoprotein samples (see Figure 4) strongly indicate that *N*-glycans from *P. margaritaceum* commonly contain Xyl and Fuc residues. 

### 3.6. The P. margaritaceum Secretome and N-Glycosylation Patterns Are Closely Related to That of Embryophytes

The proteins identified in the *P. margaritaceum* secretome were used in a BLAST search against the proteome sequences of *A. thaliana*, *V. carteri*, and *C. reinhardtii*. Most of the proteins identified in *P. margaritaceum* had homologs encoded in the *A. thaliana* genome. However, in contrast, we only observed 17 and 16 protein homologs of the *P. margaritaceum* proteins in *V. carteri* and *C. reinhardtii*, respectively (see Table S5). The *P. margaritaceum* disulfide isomerases (Q9FEG4 and E3W9C1) have functionally annotated homologs in *A. thaliana* (Q9XI01 and O22263), *V. carteri* (Q9SBN2 and Q9SBN2), and *C. reinhardtii* (O48949 and A8IHI1, Table S5) while only one homolog of the proteases identified in *P. margaritaceum* was found in *V. carteri* (D8U6V0) and *C. reinhardtii* (A8I5R9). Even though these proteins from *V. carteri* and *C. reinhardtii* have not yet been functionally annotated, they are predicted to contain a domain of the papain family of cysteine proteases. Similarly, the homologs of LRX1 (Q9LJ64) found in *V. carteri* (D8UG11) and *C. reinhardtii* (A8J9P5) were computationally predicted to contain a leucine-rich repeat domain (LLR_8). In contrast, FLA (G7ILU2), superoxide dismutase (G9M4K4), GDSL esterase/lipase (Q9M2R9), a probable endo-1,3(4)-beta-glucanase (A2QBQ3), and a root cap protein (B6TV36) did not have homologs in either *V. carteri* or *C. reinhardtii* (Table S5). 

**Figure 5:**
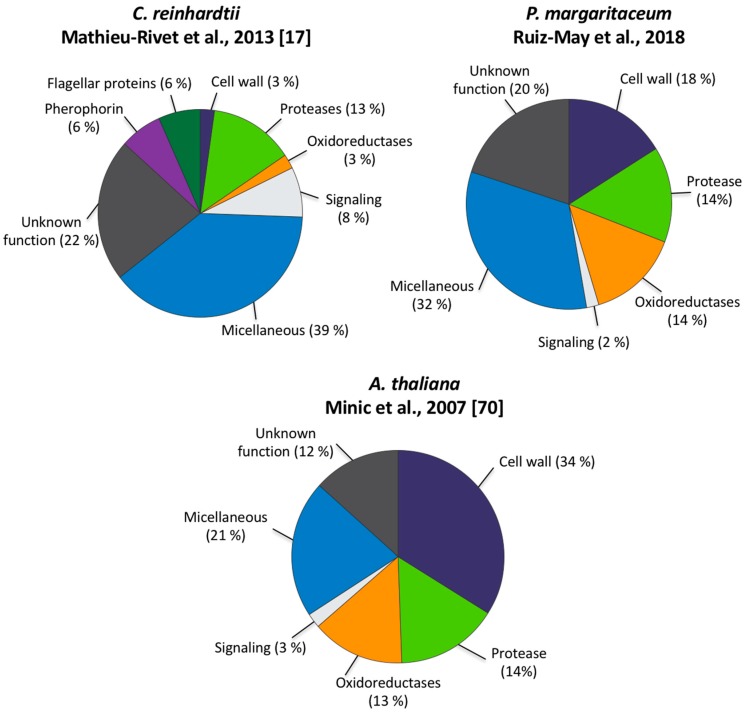
Comparison of the secretomes of *Chlamydomonas reinhardtii*, *Penium margaritaceum*, and *Arabidopsis thaliana*. Secreted proteins analyzed in the *C. reinhardtii* and *A. thaliana* secretome were enriched using *N*-Glyco-filter-aided sample preparation (FASP)-ConA and ConA-based lectin affinity chromatography (LAC), respectively [17,70]. These proteins were functionally classified using the Blast2Go software (www.blast2go.com) and UniProt functional annotation (http://www.uniprot.org/), according to Jamet et al. [62].

Comparative analyses of the secretome of *C. reinhardtii* determined with *N*-Glyco-filter-aided sample preparation (FASP)-ConA [17] and those of *P. margaritaceum* and *A. thaliana* obtained by LAC-ConA [70], revealed striking differences in the proportion of cell-wall associated proteins (see Figure 5). The *C. reinhardtii* secretome contained a small proportion of predicted cell wall associated proteins (3%) compared to *P. margaritaceum* and *A. thaliana*, which had 18% and 34%, respectively (see Figure 5). Proteins classified as oxido-reductases and proteins associated with signaling were observed in a similar proportion in the secretome of *P. margaritaceum* and *A. thaliana* (see Figure 5) while proteins functionally classified as proteases were present in similar proportions in all secretomes analyzed in this study (see Figure 5). Our data suggests that *P. margaritaceum* and embryophytes not only share similar cell wall structural features [37] but also have similar secretomes. 

**Figure 6:**
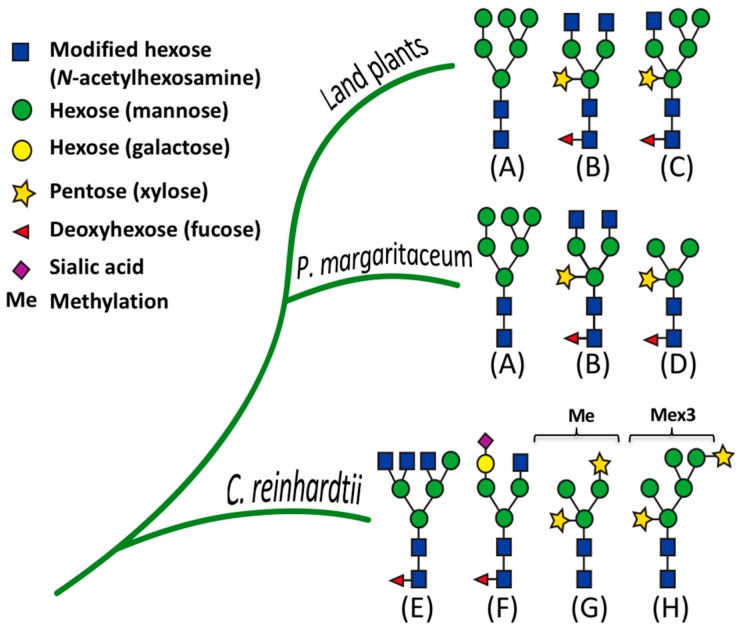
Comparison of the structures of *N*-glycans identified in *Chlamydomonas reinhardtii*, *Penium margaritaceum*, and land plants (embryophytes). (**A**) high mannose, (**B**) complex, (**C**) hybrid, (**D**) paucimannose, (**E**) tetra-antennary, (**F**) sialylation. (**G**,**H**) correspond to *N*-glycans with an additional xylose residue attached to the outermost methylated mannose residues.

We further analyzed our results by comparing the structural features of *N*-glycans in *P. margaritaceum* to those in *C. reinhardtii* [29] and land plants [70] based on the MS analysis (see Figure 6 and Figure S2). The *N*-glycans of *P. margaritaceum* were found to contain sugar residues similar to those found in the *N*-glycans of embryophytes such as the β-1,2-xylose and α-1,3-fucose residues. In *P. margaritaceum*, we determined the presence of high-mannose, complex, and paucimannose structures (see Figure 6) with the frequent occurrence of β-1,2-xylose and α-1,3-fucose residues in the complex and paucimannose structures. Recent studies indicated that *C. reinhardtii N*-glycans contain β-1,2-xylose residues and that the α-1,3-fucose residues are present in small proportions and carry one additional xylose residue on the outermost mannose residues [17] (see Figure 6). Additionally, structural features including the tetra-antennary structure and the sialic acid residues characteristic of the mammalian *N*-glycans are present in the structures of the *N*-glycans of *C. reinhardtii*. Since we did not detect these in *P. margaritaceum* (see Figure 6), our data are consistent with the *P. margaritaceum* secretome and *N*-glycosylation patterns being more closely related to those of embryophytes.

## 4. Discussion

### 4.1. Proteomic Analysis of P. margaritaceum Using an RNA-Sequence Database as Reference

Extensive and accurate genome sequence information provides a platform for effective proteome profiling studies and this has previously limited the application of proteomic technologies to model plants with sequenced genomes. However, the increasing availability of cDNA sequences from different organisms as well as next generation sequencing are providing access to transcriptome information at a rapidly declining cost [82,83]. This has expanded the options for systematic identification of proteins even in species with only draft genome sequences and those with no genomic information at all. In our present study, we were able to identify more proteins using a *P. margaritaceum* RNA-Seq based database rather than by searching the PlantGDB. This is consistent with previous studies reporting the value of a species-specific database for protein identification [82,84,85,86]. However, the numbers of identified peptides per protein were low in our analyses. It is known that secreted proteins are heavily decorated with multiple post-translational modifications (PTMs) including different types of glycosylation (*N*-linked and *O*-linked, *C*-mannosylation, and glycosylphosphatidylinositol anchors) [87,88] and the *P. margaritaceum* secretome may contain multiple PTMs that could increase the complexity of mass spectra, which resulted in fewer matches. However, future studies will help determine whether a more complete DNA sequence database will further enhance protein identification.

We carried out an in silico analysis of the subcellular location of identified proteins in *P. margaritaceum* using four different bioinformatic tools including Signal P [63], PSORT [64], Target P [65], and PredAlgo [66]. The latter was designed specifically for prediction of protein subcellular location in green algae [66]. This software showed significant improvements in the capacity to discriminate between chloroplast and mitochondrial proteins, but it did not enhance the signal peptide prediction compared to other prediction programs [66]. However, using PredAlgo for the prediction of subcellular proteins in *P. margaritaceum* resulted in fewer hits (see Table S1). In addition, well-known cell wall proteins such as FLA (G7ILU2) and GDSL esterase/lipase (Q9M2R9) were predicted to be chloroplast localized (see Table S1). We, therefore, used SignalP 4.1 [63] to predict the presence of signal peptides and PSORT [64] as well as Target P [65] for protein subcellular location of identified protein sequences. Most of the proteins enriched by ConA affinity were predicted to be secreted (see Figure 2), which is in agreement with previous report [30] and highlights the value of LAC as a strategy for secretome analysis.

### 4.2. The Secretome of P. margaritaceum is Closely Related to that of Embryophytes

LAC has been used in several analyses of the secretome of land plants [30,59,70]. In this study, we identified several cell wall associated proteins in the *P. margaritaceum* secretome that have also been found in studies of embryophyte cell walls using LAC (see Table 1). We used monoclonal antibodies against land plant AGP glycans and extensin to verify that these types of proteins are present in not only the *P. margaritaceum* cell wall but also in the EPS. Interestingly, immunodetection of extensin epitopes (JIM20, Figure 3E) showed the presence of multiple transverse bands on the cell wall, which is similar in appearance to banding patterns observed in *P. margaritaceum* cells labeled with ConA [41]. Furthermore, these bands resemble the bands of pectic homogalacturonan (HG) that are present during expansion of the cell wall of pollen tubes in land plants [89,90] and we hypothesize that there may be an interaction between extensin and pectin, which is suggested for sugar beet cell walls [91]. Immune-detection of AGP epitopes showed a dense punctuation in the cell wall and EPS (see Figure 3A–D), which is in agreement with studies in *A. thaliana* where two AGP protein isoforms were identified in both the culture media and cell wall of suspension culture cells [73]. Another interesting finding from this previous study was that the *O*-glycans of the AGPs were covalently cross-linked to the matrix of hemicellulose and pectic polysaccharides. This close association between AGPs and cell wall polymers was suggested to explain the modification of cellulose deposition and biochemical properties of the cell wall when AGP gene expression was altered [92,93,94,95]. It is possible that similar processes occur in *P. margaritaceum*.

Taken together, the identification of proteins known to be present in the cell wall of land plants (see Table 1), localization of AGP and extensin epitopes in the wall and EPS of *P. margaritaceum* (see Figure 3), and comparative bioinformatic analyses of green plant taxa (see Figure 5 and Table S4) suggests that the cell walls of *P. margaritaceum* and land plants have considerable structural and functional similarities. Similarities in their secretomes are also apparent. For example, proteases and proteins related to oxidation-reduction were found in similar proportions in the secretomes of *P. margaritaceum* compared to *A. thaliana* (see Figure 5). We note that protein disulfide isomerase S-2 (E3W9C1), protein disulfide isomerase-like 1-3 (Q8VX13), and endoplasmin homolog (Q9STX5) have a predicted localization in the ER and have annotations related to the biological processes of ‘protein folding’ (GO:0006457) and ‘response to endoplasmic reticulum stress’ (GO:0034976). Although some of the proteins we identified such as E3W9C1 are not predicted to be extracellular, their presence in the secretory system provides a pathway by which they might may ultimately exit the cell as part of the massive secretory process/bulk flow that is evident in *P. margaritaceum.* However, we note that cell lysis cannot be ruled out so these data await confirmation using orthogonal techniques.

### 4.3. The EPS is Tightly Associated with the Cell Wall in P. margaritaceum

It has been suggested for several organisms that the EPS plays an important role in biofilm development, adhesion, gliding, and interaction with other organisms and protection against desiccation [39,40,47,96]. *P. margaritaceum* produces significant amounts of EPS, which covers the outer surface of the cell and compositional analyses of the EPS in *P. margaritaceum* showed similar monosaccharides to those found in the cell wall of land plants in an atypical ratio [39,96]. Molecular characterization of the EPS of bacteria and other microorganisms has identified not only polysaccharides but also proteins, nucleic acids, and lipids [47]. In agreement with earlier studies, we detected proteins that are entirely extracellular as well as those that have domains that are located in the plasma membrane (see Table 1) and the cytosol (see Table S3) in the EPS of *P. margaritaceum*. To highlight one example, formin-like protein 2 (Q7XUV2) has been associated with actin-related processes such as actin nucleation, which polarized cell expansion and signaling [97,98]. It was shown that formin 1 from *A. thaliana* (AtFH1) forms a bridge from the actin cytoskeleton across the plasma membrane and that its extracellular domain is anchored in the cell wall [71]. These interconnections may be important in actin remodeling during cell growth or expansion or under stress conditions [97,98]. In addition, it was recently suggested that AtFH14 might regulate actin dynamics through association with profilin [98]. Interestingly, we also identified profilin proteins (Q9XF40 and Q95VF7, Table S3) in the EPS fraction. The detection of both formin-like protein 2 and profilin proteins is consistent with a similarly close association between the cytoskeleton and extracellular matrix of *P. margaritaceum*.

### 4.4. N-Glycosylation Patterns Are Conserved between P. margaritaceum and Land Plants 

*N*-Glycosylation is widespread in different kingdoms with multiple *N*-glycan structural variants [24]. However, their structural variation during plant evolution is not well understood. Given that the CGA are located at a pivotal evolutionary point, we hypothesized that an analysis of the *P. margaritaceum* glycoproteome could provide insights into the evolution of *N*-glycosylation. We found that the *N*-glycans from *P. margaritaceum* have identical structural features to those of land plants and that their structure corresponded to the high mannose, complex, and paucimannosidic types containing β-1,2-xylose (Xyl) and α-1,3-fucose (Fuc) residues linked to the core Man_3_GlcNAc_2_. More than two decades ago, analysis of *N*-glycans from the sexually-induced glycoprotein pheromone of *V. carteri* suggested the presence of Xyl residues and the absence of Fuc residues [99]. However, this has not yet been confirmed by high accuracy mass spectrometry analysis. *C. reinhardtii N*-glycans have Xyl and Fuc residues [17,29], but they also have features that distinguish them from land plant *N*-glycans such as the partial methylation of the external mannose residues, the presence of additional Xyl residues on the outermost Man residues, and the presence of sugar features similar to those found in *N*-glycans of mammals [17,29]. We note that most of the studies mentioned above were carried out with different mass spectrometry platforms and sample preparation strategies. For instance, previous reports have suggested the occurrence of sialic acid in the *N*-glycan structures of *C. reinhardtii* [28,29] while a more recent report found it not to be present [17]. Therefore, corroboration of particular features of *N*-glycan structures is important while taking advantage of multiple MS fragmentation methods and enrichment approaches [26,100].

We conclude that *N*-glycosylation in *P. margaritaceum* is more closely related to that of embryophytes than to that of chlorophytes and our data provide evidence of the conservation of ‘land plant-like’ *N*-glycosylation over at least the last 450 million years. Additional analysis of the *N*-glycan structures from both later diverging chlorophytes and earlier diverging charophytes will presumably further resolve the timeline of plant *N*-glycosylation evolution.

## Figures and Tables

**Table 1:** Secreted proteins identified in *Penium margaritaceum* cultures.

Unigene ID ^a^	Name	UAN ^b^	Species	PSORT ^c^	*N*-Glyco ^d^	Experiment
*Cell wall associated proteins*						
Penium13014-0R	Probable endo-1,3(4)-beta-glucanase Aspergillus niger	A2QBQ3	*Aspergillus niger*	Extr	5	ConA
PU01918-0R	Leucine-rich repeat extensin-like protein 1	Q9LJ64	*Arabidopsis thaliana*	Extr	3	ConA
Penium10692-1R	Arabinogalactan protein	Q9M7I5	*Zea mays*	Extr	0	ConA
Penium51331-0F	Fasciclin-like arabinogalactan protein	G7ILU2	*Medicago truncatula*	Extr	3	ConA
Penium05518-1F	Structural constituent of cell-wall, putative	B9S9J6	*Ricinus communis*	Extr	1	EPS
PU11354-0R	Putative uncharacterized protein (Bacterial cellulose synthase subunit)	E6TGN8	*Mycobacterium sp.*	Ext	5	ConA
PU02505-0R	Formin-like protein 2	Q7XUV2	*Oryza sativa subsp. japonica*	Plas	2	EPS
*Proteolysis*						
corb_UMD_Coleochaete_c15397_c_s	Peptidase S1 and S6	A2Q336	*Medicago truncatula*	Extr	3	ConA
Penium21547-0F	Cysteine protease, putative	B9R8S7	*Ricinus communis*	Extr	1	ConA
Penium50713-1F	Intramembrane protease RasP/YluC	A0A0H4WXM9	*Myxococcus sp.*	Plas	1	EPS
corb_UMD_Coleochaete_c16755_c_s	Propeptide PepSY amd peptidase M4	E8WAR7	*Streptomyces flavogriseus*	Extr	1	ConA
PU17998-0F	Secreted serine protease	B1VSD5	*Streptomyces griseus subsp. griseus*	Extr	3	ConA
PU16802-1F	Cysteine proteinase 2	Q10717	*Zea mays*	Vacu	2	ConA
PU11399-1F	Membrane protease subunit, stomatin/prohibitin	K6FM44	*Desulfovibrio magneticus*	Extr	1	ConA
Penium04469-1F	Serine carboxypeptidase S28 family protein	D7MUL9	*Arabidopsis lyrata subsp. lyrata*	Vacu	6	ConA
*Oxidoreductases*						
PU21449-0R	Superoxide dismutase	G9M4K4	*Pogonatum inflexum*	Extr	0	EPS
Penium04764-0F	Ferredoxin thioredoxin reductase catalytic beta chain family	D7L839	*Arabidopsis lyrata subsp. lyrata*	Extr	0	EPS
PU02432-1F	Epimerase/dehydrogenase	D7FJ06	*Ectocarpus siliculosus*	Extr	0	EPS
Penium24287-2R	Protein disulfide isomerase	Q9FEG4	*Triticum durum*	Vacu	1	ConA and EPS
PU08835-1F	Protein disulfide isomerase S-2	E3W9C1	*Glycine max*	ER	0	EPS
PU15966-1R	Protein disulfide isomerase-like 1-3	Q8VX13	*Arabidopsis thaliana*	ER	8	ConA
PU13392-0F	Microneme protein, putative	A0A086JBX3	*Toxoplasma gondii*	Golg	8	ConA
PU14524-1R	Glutaredoxin-C4	Q8LFQ6	*Arabidopsis thaliana*	Vacu	0	EPS
*Miscellaneous*						
PU00748-1F	GDSL esterase/lipase	Q9M2R9	*Arabidopsis thaliana*	Extr	3	ConA and EPS
Penium53827-1R	Glucosidase II beta subunit	B9SBM9	*Ricinus communis*	Extr	0	ConA
Penium51452-0R	Root cap protein 1	B6TV36	*Zea mays*	Extr	1	EPS
Penium50864-2R	Alpha amylase, catalytic domain protein	A7A7M5	*Bifidobacterium adolescentis*	Chlo	8	EPS
corb_Contig1457	Protein phosphatase 2C	G7I7K2	*Medicago truncatula*	Plas	1	EPS
PU00133-0R	Endonuclease 5	F4JJL3	*Arabidopsis thaliana*	Extr	4	ConA and EPS
PU14522-2F	PXN-FBPL	Q2LK77	*Xenopus laevis*	Vacu	5	ConA
Penium15798-2R	Alpha/beta-type gliadin	Q41632	*Triticum urartu*	Vacu	0	ConA
Penium42317-1F	Pollen coat oleosin-glycine rich protein	Q6V5D9	*Olimarabidopsis pumila*	Extr	0	ConA
PU21202-1R	BURP domain-containing protein 7	Q60E34	*Oryza sativa subsp. japonica*	Extr	0	ConA
PU12863-2F	Putative ABC-type transport system	J2K1X5	*Rhizobium sp. CF080*	Extr	1	ConA
PU00769-1R	Endoplasmin homolog	Q9STX5	*Arabidopsis thaliana*	ER	3	ConA
Penium03560-1F	Serine protease inhibitor	Q32TF4	*Argopecten irradians*	Extr	1	ConA
PU08363-0R	Carbonic anhydrase	H1XWM8	*Caldithrix abyssi DSM 13497*	Extr	3	EPS
Penium02868-2F	Peritrophin-1	E2ADF6	*Camponotus floridanus*	Extr	6	ConA
PU21572-2F	Putative cuticle protein	C0H6H4	*Bombyx mori*	Extr	1	ConA
PU26029-0R	Olfactory receptor 5AK2	Q8NH90	*Homo sapiens*	Plas	2	ConA
PU32535-2R	Type III secretion protein SpaR/YscT/HrcT	A1TJC4	*Acidovorax citrulli*	Plas	1	ConA
*Unknown function*						
Penium54960-0F	Uncharacterized protein	G7ME15	*Macaca mulatta*	Plas	12	ConA
PU20188-1R	Putative uncharacterized protein	D7SHJ2	*Vitis vinifera*	Extr	0	ConA
PU23729-1F	Predicted protein	A9TWI4	*Physcomitrella patens subsp. patens*	Extr	8	ConA
PU11719-0R	Putative uncharacterized protein	D8U3A6	*Volvox carteri*	Extr	0	ConA
Penium17901-2F	Putative uncharacterized protein	B6SPS4	*Zea mays*	Plas	0	ConA
Penium42683-0F	Predicted protein	A9RQJ8	*Physcomitrella patens subsp. patens*	Extr	1	ConA and EPS
Penium15957-1F	Uncharacterized protein	L7UB03	*Myxococcus stipitatus DSM 14675*	Extr	0	EPS
PU16053-0F	Putative uncharacterized protein	A3CD48	*Oryza sativa subsp. japonica* (Rice)	Plas	0	ConA
Penium33821-0F	Putative uncharacterized	G7E6T5	*Mixia osmundae*	Plas	3	ConA
PU00167-0R	Putative uncharacterized protein	F1YT29	*Acetobacter pomorum*	Plas	3	EPS
PU08479-1R	Uncharacterized protein	L2G3N6	*Colletotrichum gloeosporioides*	ER	0	ConA

**^a^** Unigenes from in-house RNA-seq database; **^b^** UniProt accession number; **^c^** Subcellular localization prediction of proteins identified in this study were based on PSORT s (http://wolfpsort.org). Extr: extracellular, ER: endoplasmic reticulum, Plas: plasma membrane, Vacu: vacuole, Golg: Golgi apparatus, Chlo: chloroplast; **^d^** *N*-glycosylation sites were predicted using NetNGlyc 1.0 (http://www.cbs.dtu.dk/services/NetNGlyc/).

**Table 2:** Endogenous *N*-glycopeptides and *N*-glycans identified from *Penium margaritaceum* cultures.

Unigene	Protein	UAN ^a^	E-Value	Species	Peptide	Pep Mass [M + H]^+^	*N*-Glycan Structures ^b^
PU00576-0F	Receptor of activated protein kinase C 1	A8J8Y1	4 × 10^−26^	*Chlamydomonas reinhardtii*	GSQLNGTYA	910.32	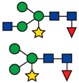
PU00894-1F	Root cap protein 2	Q9ZQT1	3 × 10^−11^	*Zea mays*	MAGNVSVVGK	961.32	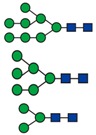
						687.52	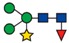
						771.48	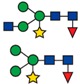
						815.40	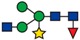
						815.40	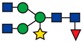
						815.40	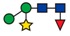
						815.48	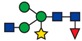

**^a^** UniProt accession number; **^b^** Glycan structures were manually determined as outlined in material and methods.

## Supplementary Materials

The following are available online at http://www.mdpi.com/2227-7382/6/2/14/s1. Figure S1: proteomic pipeline used in this study, Figure S2: MS/MS spectra of endogenous *N*-glycopeptides. The *N*-glycan structures were determined manually. The symbol nomenclature for glycan representation is according to [54], Table S1: Proteins identified following Concanavalin A enrichment using a *Penium margaritaceum* RNA-Seq translated database, Table S2: Proteins identified following enrichment with Concanavalin A using the green plant database (http://www.plantgdb.org/), Table S3: Proteins identified in the extracellular polymeric substance (EPS), using a *P. margaritaceum* RNA-Seq translated database, Table S4: Proteins identified in the extracellular polymeric substance (EPS) using the green plant database, Table S5: Proteins identified in the *P. margaritaceum* secretome used in a BLAST search against the genome database of *Arabidopsis thaliana* (http://www.arabidopsis.org/wublast/index2.jsp), *Volvox carteri* (http://genome.jgi.doe.gov/pages/blast.jsf?db=Volca1) and *Chlamydomonas reinhardtii* (http://genome.jgi-psf.org/pages/blast.jsf?db=Chlre4).
