# Supplementary figures and images for: The Secretome and N-Glycosylation Profiles of the Charophycean Green Alga, Penium margaritaceum, Resemble Those of Embryophytes
Source: Proteomes. 2018 Mar 21;6(2):14. doi: 10.3390/proteomes6020014 (PMC6027541; doi:10.3390/proteomes6020014)

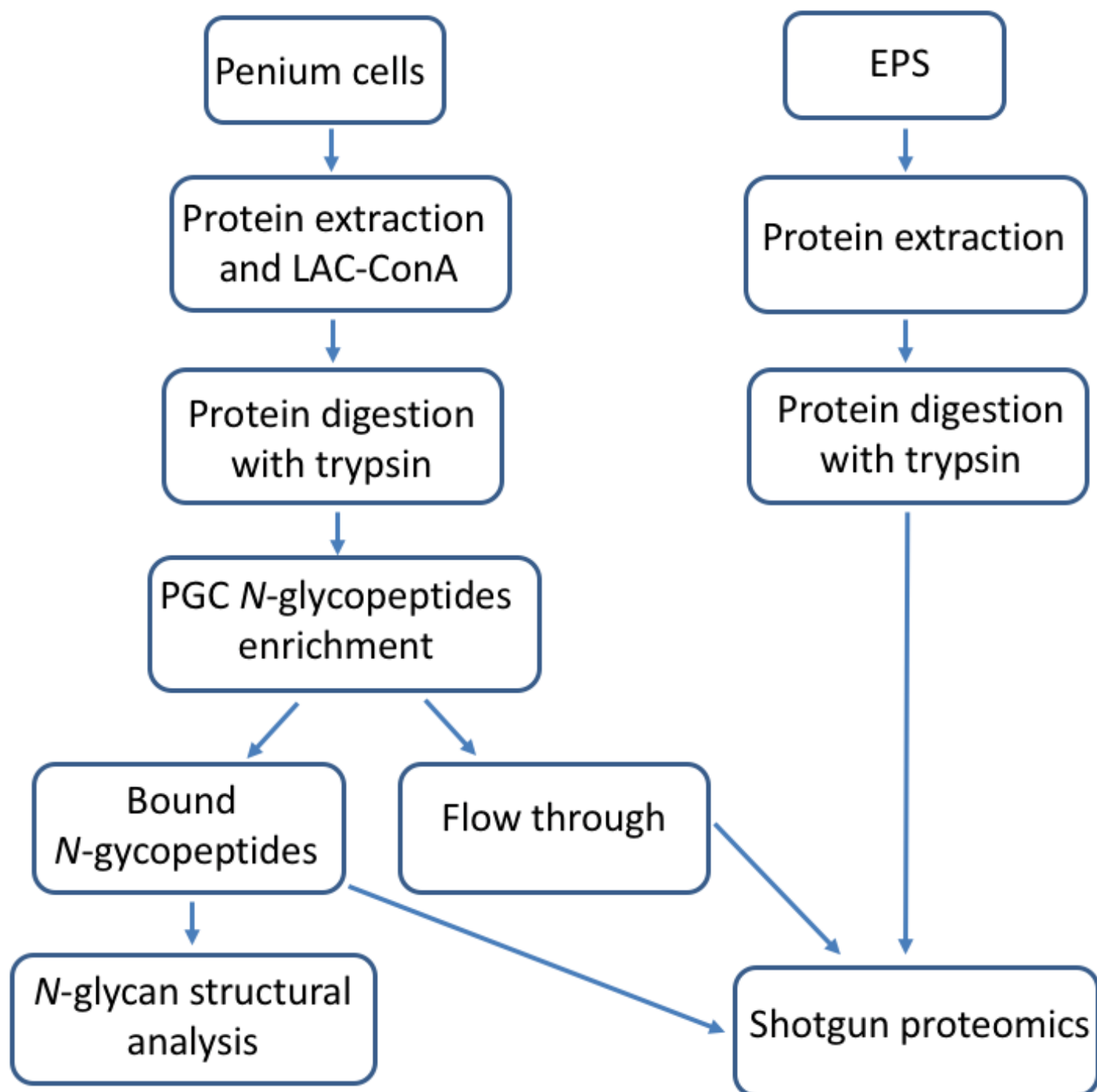

Supplement: Supplementary file 1 [file proteomes-06-00014-s001.zip › Figure S1.pdf]
